# The Stomach Capacity is Reduced in Intrauterine Growth Restricted Piglets Compared to Normal Piglets

**DOI:** 10.3390/ani10081291

**Published:** 2020-07-28

**Authors:** Julie C. Lynegaard, Janni Hales, Marlene N. Nielsen, Christian F. Hansen, Charlotte Amdi

**Affiliations:** 1 Department of Veterinary and Animal Sciences, Faculty of Health and Medical Sciences, University of Copenhagen, DK-1870 Frederiksberg C, Denmark; julie.lynegaard@sund.ku.dk (J.C.L.); jhp@skiold.com (J.H.); mann@seges.dk (M.N.N.); cfha@seges.dk (C.F.H.); 2 SKIOLD JYDEN, Idomvej 2, 7570 Vemb, Denmark; 3 Pig Research Centre, Danish Agriculture and Food Council, Axeltorv 3, DK-1609 Copenhagen V, Denmark

**Keywords:** colostrum supplement, intrauterine growth restriction, newborn, piglet, stomach capacity

## Abstract

**Simple Summary:**

Large litters have resulted in a higher percentage of piglets suffering from intrauterine growth restriction (IUGR). There is a higher mortality in this subset of piglets and a large number die because they do not receive enough nutrients for energy within the critical first 24 h after birth. One source of nutrients for energy could be supplementation with extra colostrum from previously milked sows. However, there is no knowledge on the stomach capacity of IUGR piglets, and therefore, of how much colostrum could potentially be supplemented. This is important information in order to recommend how much supplementary colostrum IUGR piglets need in order to survive.

**Abstract:**

Selection for increased litter sizes have decreased the average birth weight of piglets and up to 30% of newborn piglets in Danish herds show signs of intrauterine growth restriction (IUGR). It has been reported that around 48% of liveborn piglets dying between birth and weaning have empty stomachs, and that IUGR piglets do not ingest the recommended amount of colostrum to survive. The aim of this study was to investigate how much colostrum could be administrated depending on whether they were IUGR compared to normal piglets. Seventy-two piglets within 24 h of farrowing were classified as either IUGR or normal based on their head morphology. Stomach weight, length and capacity were measured along with bodyweight (BW). The results displayed a decreased BW, empty stomach weight and capacity in IUGR piglets, as well as a decreased relative stomach capacity in IUGR compared with normal piglets. In conclusion, birth weight is not the only factor influencing stomach capacity, and IUGR piglets have a smaller stomach capacity compared with normal piglets. It is estimated that IUGR piglets have the capacity to be given a bolus of 25 mL per kg/BW, whereas a normal piglet have a higher capacity (30 mL per kg/BW).

## 1. Introduction

The hyperprolific modern sow has resulted in an increased litter size as well as a decrease in individual piglet birth weight, with up to 30% of newborn piglets in Danish herds showing signs of intrauterine growth restriction (IUGR) [1,2,3]. Intrauterine growth restriction is usually defined as an impaired growth during prenatal development [4], and has a negative effect on postnatal growth and pre-weaning survival [3,5,6]. Piglets subjected to IUGR have higher nutritional demands, in the form of energy per kg/bodyweight (BW), due to a higher surface-to-volume ratio [7,8]. Additionally, IUGR piglets have a smaller stomach at birth due to a lower BW [9,10]. Furthermore, IUGR piglets have a higher mortality within the first 24 h postpartum [2], and it is therefore paramount that these piglets receive additional care and/or nutritional supplements to increase their survivability.

Typically, using nurse sows suitable for rearing IUGR piglets could be one management strategy. However, due to increasing litter sizes, up to 40% of sows in a herd are used as nurse sows [11]. Although this removes litter competition [12] an insufficient intake of colostrum is one of the main underlying factors affecting early deaths in piglets [13,14]. Therefore, more intense/laboursome management strategies are possibly needed. Early colostrum intake is vital for the newborn piglet, in order to meet their nutritional requirements for thermoregulation, physical activity and growth [8,15], and in providing antibodies for immune protection [16]. A previous study found that approximately 48% of liveborn piglets that died between birth and weaning had an empty stomach [2], whereas another study concluded that 72% of piglets dying within four days of farrowing had not received any colostrum [17]. This suggests that the nutritional intake of these neonatal piglets is inadequate. A study reported that normal piglets should ingest at least 200 g of colostrum within the first 24 h in order to survive [16], however, it has been estimated that IUGR piglets only ingest about half the recommended colostrum [1], although they have a gastric emptying rate similar to normal piglets [18].

Since IUGR piglets are smaller in size and have suffered from an impaired prenatal growth, they are expected to have a lower stomach capacity than normal piglets due to the rapid growth and maturation of the gastro-intestinal tract in the weeks before birth [19]. However, there is little knowledge on the stomach size and capacity of IUGR piglets around birth, and consequently how much supplemental colostrum could be given to increase their survivability and growth. The stomach capacity of newborn IUGR piglets was therefore investigated. We hypothesised that IUGR piglets have a smaller stomach capacity than normal piglets and should therefore have different recommendations regarding colostrum supplements within the first 24 h postpartum.

## 2. Materials and Methods

### 2.1. Ethical Approval

The experiment was carried out with approval from the Danish Experimentation Inspectorate, j.nr. 2016-15-0201-00894.

### 2.2. Animals and Experimental Design

The experiment was conducted with euthanised piglets from two commercial Danish piggeries, both with sows (Danish Landrace × Danish Yorkshire) inseminated with Duroc semen (Hatting KS, Horsens, Denmark). A total of 72 piglets (23 normal and 49 IUGR) were used in this study, however, two IUGR piglets had to be excluded from the study, due to a rupture of the stomach. The piglets were given a visual classification of either IUGR or normal based on their head morphology (modified after [2,20]). Briefly a piglet was given a score ranging from normal to severe IUGR, recognizing the IUGR piglet by: (1) a steep dolphin-like forehead, (2) bulging eyes, and (3) hair with no direction of growth. The piglets were classified as IUGR if two or three of the characteristics were present, and if none of the characteristics were present, the piglets were considered as normal.

Forty-four piglets (34 IUGR and 10 normal) were selected within 8 h of birth, and were intramuscularly anaesthetised with a Zoletil mix (Zoletil 50, Virbac, Kolding, Denmark), containing xylacin (Narcoxyl 20mg/mL, MSD Animal Health, Ballerup, Denmark), ketamine (Ketaminol 100 mg/mL, MSD, Animal Health, Ballerup, Denmark) and butorphanol (Torbugesic 10 mg/mL, ScanVet, Fredensborg, Denmark) and left covered in a pen filled with straw to achieve deep anaesthesia. Afterwards, the piglets were euthanised with an intracardial injection of 2–3 mL pentobarbital (200 mg/mL). The remaining 28 piglets (15 IUGR and 13 normal) died within 24 h of farrowing from natural causes, and were collected by the staff for our investigation. Piglets were excluded from the study if there were any visible signs of a ruptured gastro-intestinal tract, or if they were severely damaged by the sow and it would not be possible to collect the stomach. In addition, the body weight (BW) of the piglets was recorded.

### 2.3. Stomach Measurements

The stomach was removed from the piglet and flushed gently with water to empty out any gas and/or feed residues. Next, the stomachs were weighed on a precision scale (Radwag, Radom, Poland), and then were laid flat and their length was measured from the anterior portion to the posterior portion of the stomach using a tape measure. Afterwards, the stomachs were closed off with a metal clip by the pylorus and filled with water using a 12 mL syringe through the pars oesophagus. In order to get an estimate of the volume of the stomach, it was filled with water until the stomach began overflowing. The amount of water filled into the stomach was recorded by measuring the weight of the water-filled stomach (in a bowl to preserve the weight of the water) on a scale.

### 2.4. Calculations and Statistical Analysis

All data were analysed using the statistical software SAS (SAS Inst. Inc., Cary, NC, USA) with piglet as the experimental unit. Stomach capacity was calculated as the difference in weight between the water-filled stomach and the empty stomach. Moreover, the relative stomach capacity and relative empty stomach weight were calculated as weight/capacity per kg BW. Pearson correlations were calculated using the CORR procedure to identify relationships between BW, stomach weight, stomach capacity, stomach length and relative stomach capacity. Data on BW and stomach characteristics were normally distributed and analysed in a linear model using the GLM procedure. All interactions were tested, and were deemed non-significant with *p* > 0.05 and excluded from the model. A probability of *p* < 0.05 was considered significant and *p* < 0.10 a tendency.

## 3. Results

A total of 23 normal and 47 IUGR piglets were included in the study, with an average BW of 1268 g for normal piglets and 688 g for IUGR piglets (*p* < 0.001). Table 1 displays results from the measurements of stomach weight, capacity and length for IUGR and normal piglets.

**Figure 1:**
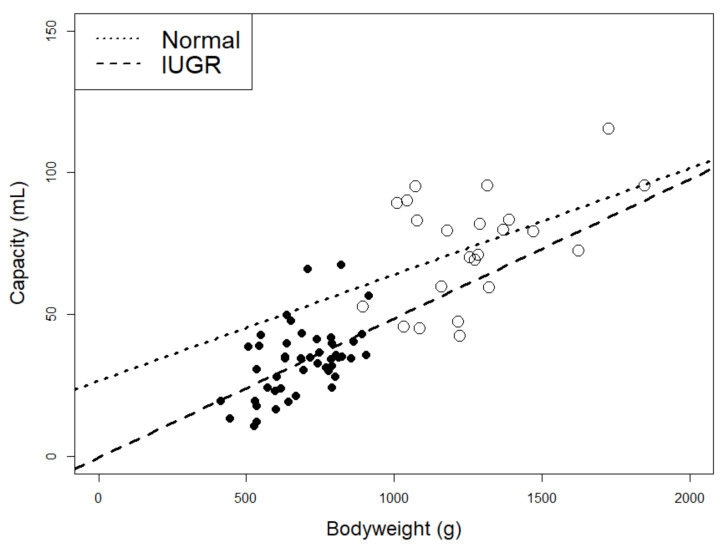
Data plotted against each other, normal piglets (white) and IUGR piglets (black). Linear regression trend lines were made for normal piglets (dotted) and IUGR piglets (broken line).

The results revealed a large variation in stomach capacity, for both IUGR (10.8–67.5 mL/kg BW) and normal piglets (34.7–88.8 mL/kg BW, Figure 1). Increasing BW of piglets increased empty stomach weight (*p* = 0.003), stomach length (*p* = 0.313) and stomach capacity (*p* = 0.008). When BW was accounted for, IUGR piglets had a smaller relative stomach capacity than normal piglets (*p* = 0.029).

Correlations between stomach characteristics are shown in Table 2. Stomach weight, length and capacity were positively related to BW (*p* < 0.001), and they were further positively correlated with each other (*p* < 0.001).

## 4. Discussion

Large litters have resulted in an increased percentage of IUGR piglets and these piglets need alternative management strategies in order to survive. In the current study the stomach capacity of an IUGR piglet both in mL and per kilo BW was found to be smaller than for normal piglets, and our hypothesis was therefore confirmed. In addition, a large variation was found for the stomach capacity of both IUGR piglets and normal piglets around birth. The stomach capacity can be increased by 50% when under pressure [21], hence there may be a difference in the capacity of the stomach between piglets having received colostrum and the ones that did not.

The results presented in this study are consistent with a pilot study studying the stomach capacity of IUGR piglets [22]. In addition, the relative stomach capacity was also significantly different between IUGR and normal piglets, which confirms the results from a previous study [18]. Other authors have reported a difference in the capacity of the stomach due to the difference in BW [10] and a difference in the stomach weight between normal and IUGR piglets [1,3]. The results from this study suggest that the stomach capacity is not only correlated with the birth weight of the piglet, but is also correlated with whether or not the piglets suffer from IUGR.

Birth weight is a major determinant of piglet vitality and ability to stimulate the udder in order to extract colostrum from teats [23], because heavier piglets may have a competitive advantage over the smaller ones for colostrum access. Moreover, there has been reported a positive correlation between birth weight and colostrum intake [24]. However, the large variation in stomach weight and capacity for both IUGR and normal piglets in the current study, suggest that other factors also influence capacity. A previous study also reported that IUGR piglets may have a lower nutrient absorption, due to fewer microvilli in the intestine [10]. This is supported by another study, demonstrating that the intestinal nutrient absorption surface was impaired in IUGR piglets for the first couple of days after farrowing [25]. Consistenly, Amdi et al., [18] also discussed that even though IUGR piglets have almost fully developed organs, they may lack in developmental maturation and this may affect their metabolism negatively.

Piglets were not systematically collected at birth in this study. This was in order to correspond to when personal in a Danish piggery would check whether any new born piglets required additional supplementation with colostrum. Our study set up therefore does not distinguish between piglets being alive for one hour or 24 h. Consequently, the stomach would have had time to grow in some of the piglets, as the stomach size increases by up to 27% within the first 24 h [26]. The stomach grows disproportionally faster than the entire body of the piglets, and some of the age differences might be causing the variation in stomach size and capacity between the different groups of piglets in this study. In order to make exact measurements on the newborn piglets’ stomach size and capacity, pigs would have to be sacrificed and the stomach removed right after parturition.

Previous studies concluded that piglets require above 200 g of colostrum within 24 h of birth to reduce mortality rate [15,16]. Whereas, Muns et al. [19] discussed, that supplementing piglets with 15 mL colostrum was enough to ensure a proper level of immunoglobulins until day four [27]. Consequently, a piglet should receive between 100–150 mL colostrum in order to gain passive immunity, but 200 mL colostrum within the first 24 h to decrease the pre-weaning mortality. Moreover, it has been reported that IUGR piglets only naturally ingest about half of the recommended amount of colostrum [1]. It therefore seems imperative to provide IUGR piglets with orally administered colostrum during the first 24 h, in order to increase their survival.

The results indicate that the stomach of a newborn IUGR piglet can contain approximately 50 mL per kg/BW. However, it must be emphasised that this is an artificial situation, where the stomachs were pumped completely full with water in order to measure the maximum capacity. In practise, it is questionable whether the piglet can cope with a completely filled stomach, or if it would potentially inflict permanent damage or make the piglets vomit. Therefore, we suggest providing IUGR piglets with about half of their maximum stomach capacity and to allocate approximately 25 mL colostrum per kg/BW. Normal piglets can be allocated a higher amount of supplemental colostrum, of about 30 mL per kg/BW. Further studies on the true digestibility and capacity are warranted. The colostrum should be provided with a feeding bottle, and not through tube feeding. By letting the piglet suckle on the bottle, the risk of both overfeeding the piglet, and rupturing the oesophagus, decreases.

Management interventions are neccesary in order to increase survival in IUGR piglets. Other studies have investigated more energy-dense supplementation and concluded that a single oral dose of fat-based energy at birth were not enough to improve growth and survival in low birth weight piglets [28]. Other researchers have tried different interventions such as glucose or colostrum without convincing results [12,27] and it is therefore questionable if it is the quality or the quantity that has an impact. Our suggestion of filling the stomach to about half at each feeding (25 mL kg/BW) is perhaps airing on the cautious side, and a consequence is that the procedure would need to be repeated several times to ensure enough colostrum to gain energy and immunity for survival. At the same time, the estimated stomach capacity and recommendations from this study are only applicable within the measured BW area of IUGR piglets (413–914 g). On the other hand, research suggests that just giving piglets one supply of colostrum does not make a difference to energy levels during the first 8 h [29]. In addition, gastric emptying rate is similar between IUGR and normal piglets, where the stomach is almost empty after around 120 min [18]. Therefore 25 mL kg/BW colostrum could be provided several times within a shorter time intervalto ensure proper antibody levels and energy supply. However, more research is needed on this matter, before a general recommendation can be implemented. Strategies involving the use of nurse sows suitable for small newborn piglets together with warmth [30] could be one solution to overcome this time-consuming issue.

## 5. Conclusions

In conclusion, birth weight is not the only factor influencing stomach capacity, and IUGR piglets have a smaller stomach capacity compared with normal piglets. It is estimated that IUGR piglets have the capacity to be given a bolus of 25 mL per kg/BW, whereas normal piglets have a higher capacity (30 mL per kg/BW).

## Figures and Tables

**Table 1:** A comparison between normal and intrauterine-growth-restricted (IUGR) piglets for different measurements of the stomach and bodyweight (BW). Values are presented as means ± SE.

	Normal	IUGR	*p*-Value
*n*	23	47	
BW (g)	1268 ± 48.4	688 ± 18.5	<0.001
Empty stomach weight (g)	7.3 ± 0.28	4.3 ± 0.21	0.003
Empty stomach weight per kg BW (g/kg)	6.0 ± 0.28	6.4 ± 0.26	0.251
Stomach length (cm)	7.0 ± 0.18	5.6 ± 0.12	0.313
Stomach capacity (mL)	74.1 ± 4.12	33.4 ± 1.79	0.008
Stomach capacity kg/BW (mL/kg)	59.1 ± 3.39	48.5 ± 2.36	0.029

**Table 2:** Correlations between stomach characteristics.

	BW	ESW ^1^	SL ^1^	SC ^1^	SCBW ^1^
BW ^2^	**-**	**0.75**	**0.68**	**0.82**	0.22
ESW ^2^		*-*	**0.69**	**0.79**	**0.48**
SL ^2^			*-*	**0.79**	**0.54**
SC ^2^				**-**	**0.71**
SCBW ^2^					-

^1^ Significance levels: bold < 0.001. ^2^ Key to acronyms: BW = body weight, ESW = empty stomach weight, SL = stomach length, SC = stomach capacity, SCBW = stomach capacity per kg BW.
